# Peritumoral edema resolves infrequently in surgically treated patients with intracranial meningioma– a retrospective study of 279 meningioma patients

**DOI:** 10.1007/s11060-025-04964-8

**Published:** 2025-03-06

**Authors:** Joonas Laajava, Mika Niemelä, Miikka Korja

**Affiliations:** Department of Neurosurgery, Helsinki University Hospital, Univeristy of Helsinki, Haarmaninkatu 4, P.O. Box 320, Helsinki, 00290 Finland

**Keywords:** Edema, Follow-up, Gliosis, Meningioma, Neurosurgery, Resection

## Abstract

**Background:**

The resolution of peritumoral brain edema (PTBE) following surgery for intracranial meningioma (IM) is poorly understood. We hypothesized that PTBE represents a more permanent rather than resolving parenchymal change. Therefore, our aim was to assess the frequency of PTBE resolution following gross total resection (GTR) of IM.

**Methods:**

IM patients who were operated on in the study hospital between 2000 and 2020, who had preoperative magnetic resonance imaging (MRI) showing PTBE and a follow-up MRI performed at least one year after surgery, were retrospectively identified. To minimize confounding by PTBE related to a postoperative residual tumor, only patients who had undergone GTR were included. PTBE was defined as hyperintensity on either pre- or postoperative fluid-attenuated inversion recovery (FLAIR) MRI sequences.

**Results:**

A total of 279 adult meningioma patients were retrospectively identified. Of these, 208 (74.6%) were graded as World Health Organization grade 1 and 71 (25.4%) as grade 2. Of the 279 patients who had the first postoperative follow-up MRI at one year or later, PTBE changes persisted in 270 (96.8%) patients. However, over 90% resolution in PTBE volume was observed in 102 (35.8%) patients during the median MRI follow-up of 5.0 years (2.3–6.5). Higher edema index (*p* <.001) and temporal PTBE location (*p* =.018) were associated with higher resolution percentage of preoperative PTBE.

**Conclusion:**

Persisting PTBE is a common finding following GTR of IMs. While complete resolution of PTBE is rare, considerable resolution is often seen. The nature and exact cause of these persisting parenchymal changes are unclear, but they likely represent gliosis.

**Supplementary Information:**

The online version contains supplementary material available at 10.1007/s11060-025-04964-8.

## Introduction

Intracranial meningiomas (IMs) are the most common primary extraparenchymal intracranial tumors [1]. Over 70% occur in women, and the incidence increases with age [1]. The World Health Organization (WHO) classifies IMs into three grades [2]. Approximately 80% of operated IMs are grade 1 (benign), 18% are grade 2 (atypical), and 2% are grade 3 (anaplastic) [1].

Magnetic resonance imaging (MRI) is typically used for diagnosis and follow-up [3]. Preoperative peritumoral brain edema (PTBE) is present in about half of the IM patients [4–6], appearing as hyperintensity on T2-weighted and fluid-attenuated inversion recovery (FLAIR) MRI sequences [7, 8]. Despite preoperative PTBE having been linked with increased mortality rates as well as pre- and postoperative seizures [8–11], limited information is available regarding the resolution of PTBE following surgery.

According to a recent systematic review, previous studies on PTBE resolution are scarce [12]. In MRI follow-ups, up to 70% of patients have been reported to achieve complete PTBE resolution within the first postoperative year [13]. However, most previous studies have focused solely on skull base IMs [12].

To better understand the frequency and extent of PTBE resolution, we assessed PTBE resolution in a large cohort of operated IM patients. We focused on patients undergoing gross total resection (GTR) to avoid confounding from residual tumors causing persistent PTBE. Based on existing evidence, we hypothesized that most IM patients with preoperative PTBE achieve complete resolution in follow-up MRIs.

## Methods

### Ethics statements

This study was approved in 2023 by the local Ethics Committee. Patient data was pseudonymized. Due to local data privacy laws, data cannot be exported from the cloud-based cybersecure operating environment Acamedic [14], which has been designed for storing, processing, and analyzing research data of patients. We did not receive any funding for this study, and we do not report any conflicts of interest. The study was conducted in accordance with the Helsinki Declaration [15].

### Study population

All adult patients who underwent GTR of supratentorial IM at the study Hospital between 2000 and 2020 were retrospectively identified. Patient identification and study material collection were conducted using electronic patient records stored in various medical software systems, such as Uranus (CGI, Helsinki, Finland), Opera (GE Healthcare, Chicago, Illinois, United States), RADU (L-Force, Helsinki, Finland), picture archiving and communication system (PACS) [16] and Qpati (Tietoevry, Espoo, Finland). First, all patients with the International Classification of Diseases 10 (ICD-10) diagnosis [17] code D32 (benign neoplasm of cerebral meninges) were identified through the Uranus database. Next, these patients’ digital MRI images were fetched from the PACS and transferred into the Acamedic platform.

IM location was initially determined based on surgical reports, and confirmed by reviewing the MRI images. IM locations were divided into five groups: convexity, falx, parasagittal, skull base IMs, and others [18]. The group “others” included intraventricular and tentorial IMs. Anterior clinoid, olfactory groove, planum sphenoidale, and sphenoid wing IMs were considered as skull base IMs. IM laterality was classified as left, right or bilateral, with the latter indicating that the IM extended across the midline. Based on the neuropathology reports, IMs were classified into 15 histological subtypes according to WHO [2]. The location of PTBE was classified based on the brain lobule most affected, categorizing it into frontal, temporal, parietal, and occipital regions.

### Inclusion criteria

Patients were required to meet the following inclusion criteria: (1) GTR of a histologically confirmed IM, (2) first intracranial surgery, (3) pre- and postoperative MRI available (minimum of a 1.5T scanner, contrast-enhanced preoperative images, FLAIR sequences), (4) no other intracranial tumors, (5) no radiotherapy administered at any point (radiation may lead to radiation-induced PTBE-like gliosis [19]), (6) age 18 or older, (7) presence of preoperative PTBE in last MRI before surgery, (8) surgery within one year of preoperative MRI, and (9) MRI follow-up time of at least one year. Extent of resection was estimated based on surgical reports and an in-hospital head CT scan. Furthermore, if any residual or recurring IMs or other intracranial tumors were identified during the follow-up, the follow-up was discontinued.

### Volume measurements

**Fig. 1:**
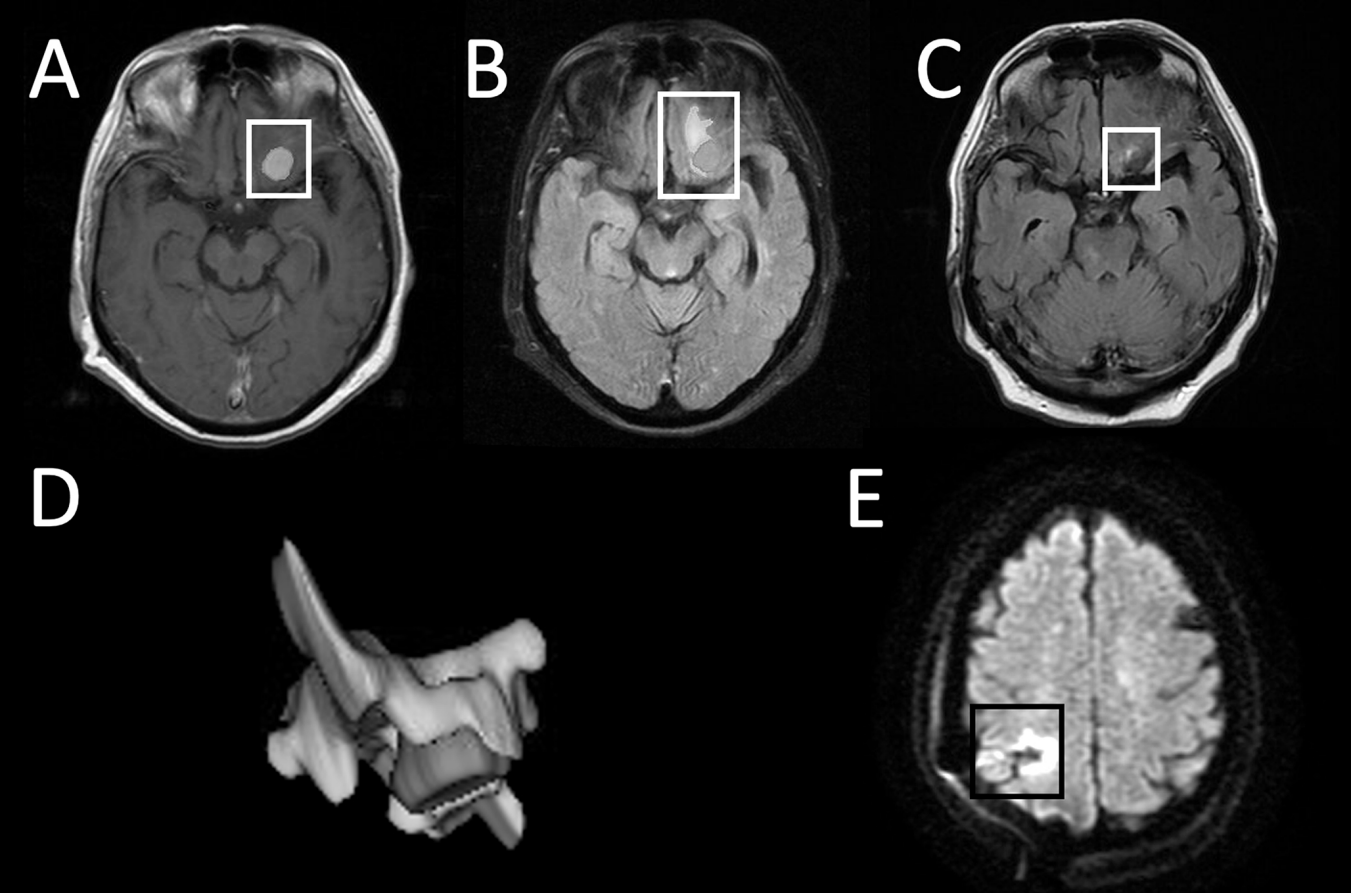
Gadolinium-enhanced T1 images were used for preoperative intracranial meningioma (IM) segmentation. Fluid-attenuated inversion recovery sequence (FLAIR) was used to segment both pre- and postoperative peritumoral brain edema (PTBE). All hyperintensity surrounding the tumor or surgical site was considered as PTBE. **(A)** T1 + C segmented IM can be seen inside the white box. **(B)** Segmented preoperative PTBE can be seen in light gray inside the white box. **(C)** Example of small FLAIR hyperintensity around the surgical site. This small change was classified as persisting PTBE (in the white box). **(D)** 3D reformat of tumor segmentation in dark gray and surrounding PTBE in light gray. **(E)** Example of acute ischemia around the surgical cavity in early postoperative in-hospital diffusion weighted MR imaging (in the black box)

IM and PTBE volume measurements were based on MRI segmentations [20]. Segmentations were done manually on MR images using the open-source software 3D Slicer (version 5.4.0) [21]. Examples of segmentations are depicted in Fig. 1. After segmentations, 3D Slicer was used to create 3D objects of the IM and surrounding edema (Fig. 1D). Volume and surface-area calculations were done automatically by 3D slicer based on segmented voxels. Edema index (EI) was calculated manually as $\:EI\:=\:\frac{Tumor\:Volume\:+\:Edema\:Volume}{Tumor\:Volume\:}$ [22].

### Follow-up protocol

According to the hospital’s follow-up protocol, patients with gross-totally resected WHO grade 1 IMs underwent MRI scans at two, five, and 10 years after surgery. Those with gross-totally resected WHO grade 2 IMs were followed up annually for up to five years; if the five-year scan showed no recurrence, a final scan was scheduled at 10 years. Exceptions to this protocol were made on a case-by-case basis, especially when patients reported new neurological symptoms. IM recurrence was assessed by reviewing all postoperative radiology reports. If a patient was found to have an IM recurrence or a new unrelated intracranial tumor on follow-up MRIs, the follow-up was discontinued.

### Intraoperative iatrogenic lesions

Intraoperative iatrogenic lesions (IILs) were assessed in patients with early postoperative in-hospital MRIs, which were performed only due to new postoperative neurological symptoms. IILs were defined as either acute ischemic lesions—new hyperintensities in diffusion-weighted imaging (DWI) sequences (Fig. 1E) [23] —or iatrogenic PTBE-like changes, which were considered as hyperintense FLAIR lesions around the resection cavity outside preoperative PTBE areas. To differentiate IILs from persisting PTBE, 3D Slicer was used to segment both. Specifically, preoperative PTBE (Fig. 1B), postoperative DWI hyperintensities (Fig. 1E), and IILs in in-hospital MRIs were segmented separately. Locations of preoperative and postoperative PTBE and IILs were assessed in follow-up MRIs to determine whether persisting changes represented PTBE or IIL.

### PTBE resolution

Given the study hospital’s follow-up protocol, we aimed to report the frequency of PTBE resolution for study patients at one and two years, as well as at last MRI follow-up after surgery. The percentage that PTBE resolved postoperatively was calculated by comparing the preoperative PTBE volume to the volume in the last follow-up MRI. The resolution percentage was calculated as: $$Resolution\>percentage$$$$= \>(1 - {{PTBE\>volume\>at\>last\>follow - up\>MRI} \over {Preoperative\>PTBE\>volume}})*100\% $$.

For patients with partial or complete PTBE resolution, we assessed whether EI was associated with PTBE resolution at last follow-up. Patients were grouped by preoperative EI into small PTBE (EI < 2), moderate PTBE (EI 2–3), and large PTBE (EI > 3).

### Statistical methods

Categorical variables were reported as counts and percentages. Normally distributed numerical data is presented as mean (standard error), while non-normally distributed data is shown as median (interquartile range). For PTBE association analyses, only patients with a reduction in PTBE volume were included, as increases are likely related to iatrogenic factors. The Welch t-test was used for two-category variables (sex, recurrence, WHO grade). For variables with more than two categories (edema location, EI, location, tumor laterality, histopathology), linear regression with the “lm” function was used to assess associations with PTBE resolution percentage, using the most frequent category as a reference. The model specification was: Y = β0 + β1 × Category1 + β2 × Category2 +…+ βn × Categoryn + ϵ. Patients with unspecified sex or histopathology were excluded from those specific analyses, but included in other analyses when appropriate. P-values below 0.05 were considered statistically significant. All analyses were conducted using RStudio version 2023.9.0.463 [24].

## Results

### Study population

**Fig. 2:**
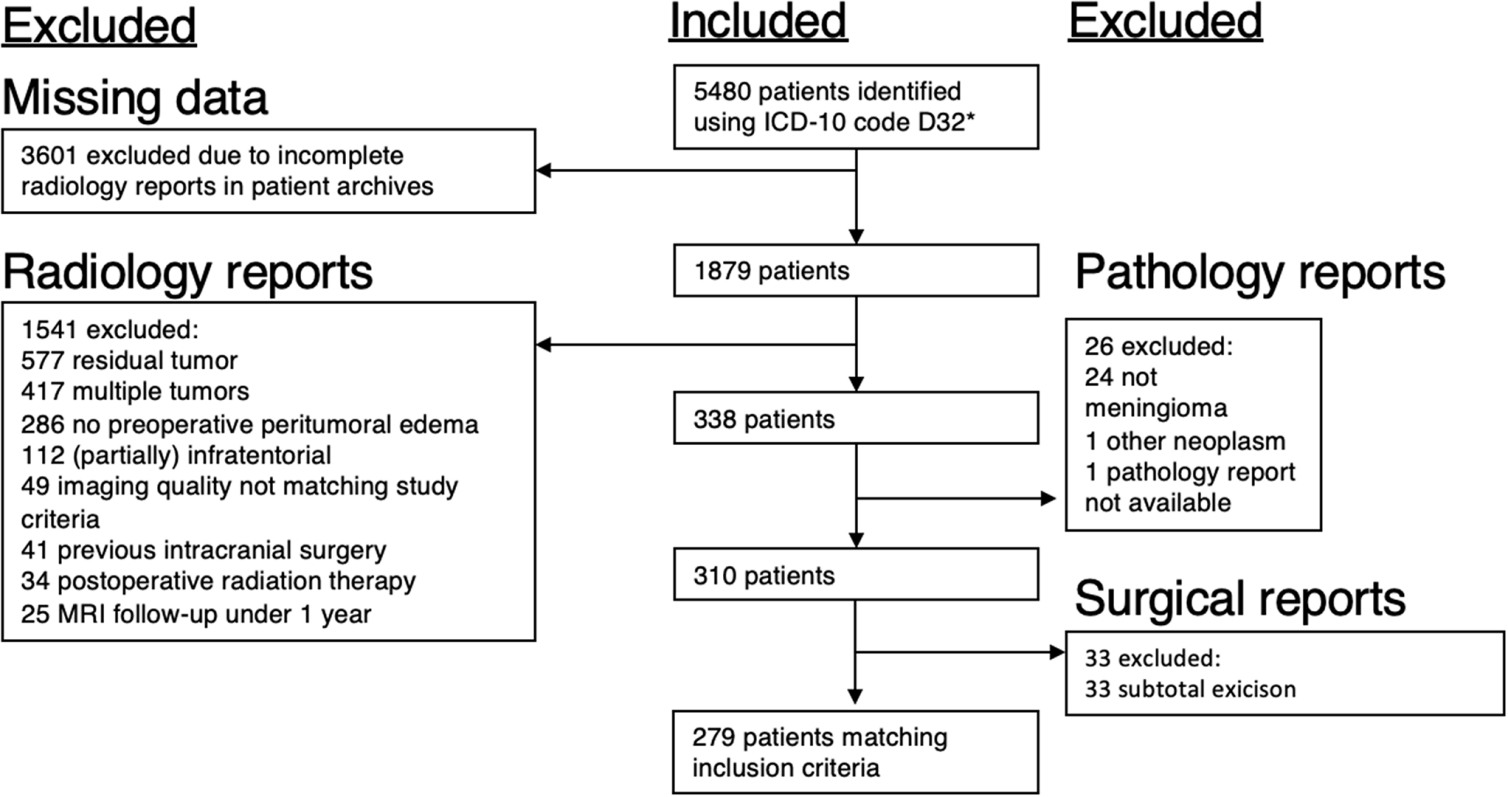
Study selection process. ICD = International Classification of Diseases, MRI = Magnetic resonance imaging, WHO = World Health Organization

Of the 5480 IM patients diagnosed between 2000 and 2020, we identified 279 IM patients who (a) showed PTBE in preoperative MRIs, (b) underwent GTR, (c) had pre- and postoperative MRI scans available, and (d) were followed-up with MRI for at least one year (Fig. 2). Patient characteristics are listed in Table 1. Angiomatous, microcystic, psammomatous, clear cell, lipomatous and metaplastic IMs were categorized into the “others” group, since each of these histopathological groups had less than 10 patients.

**Table 1:** Patient characteristics

	Overall
Overall, n (%)	279 (100%)
Age in years, median (IR)	59.0 (51.5–67.0)
Sex, n (%) Women Men Unspecified	191 (68.5%) 80 (28.7%) 8 (2.9%)
Tumor location, n (%) Skull Base Convexity Parasagittal Falx Other	97 (34.8%) 94 (33.7%) 56 (20.1%) 26 (9.3%) 6 (2.2%)
Tumor laterality, n (%) Right Left Bilateral	128 (45.9%) 121 (43.4%) 30 (10.8%)
PTBE location, n (%) Frontal Temporal Parietal Occipital	188 (67.4%) 49 (17.6%) 35 (12.5%) 7 (2.5%)
Tumor volume in cm^3^, median (IR)	20.2 (8.1–50.4)
Tumor area in cm^2^, median (IR)	41.7 (23.3–79.2)
Tumor max diameter in cm, median (IR)	4.0 (3.0-5.5)
Preoperative PTBE volume in cm^3^, median (IR)	16.4 (3.7–44.9)
Preoperative edema index, median (IR)	1.8 (1.2–3.2)
Recurrence free follow-up in years, median (IR)	5.0 (2.3–6.5)
Postoperative PTBE volume in last MRI in cm^3^, median (IR)	3.8 (0.9–9.6)
WHO Grade, n (%) I II	208 (74.6%) 71 (25.4%)
Histopathological subtype, n (%) Meningiothelial Atypical Fibrous Transitional Secretory Angiomatous Microcystic Psammomatous Clear cell Lipomatous Metaplastic Unspecified	86 (30.8%) 69 (24.7%) 38 (13.6%) 38 (13.6%) 10 (3.6%) 8 (2.9%) 5 (1.8%) 5 (1.8%) 1 (0.4%) 1 (0.4%) 1 (0.4%) 17 (6.1%)
Recurrence, n (%) No Yes Unrelated intracranial tumor	245 (87.8%) 20 (7.2%) 14 (5.0%)

PTBE = Peritumoral brain edema, IR = Interquartile range, MRI = Magnetic resonance imaging, WHO = World health organization

### Preoperative and postoperative MRIs

**Fig. 3:**
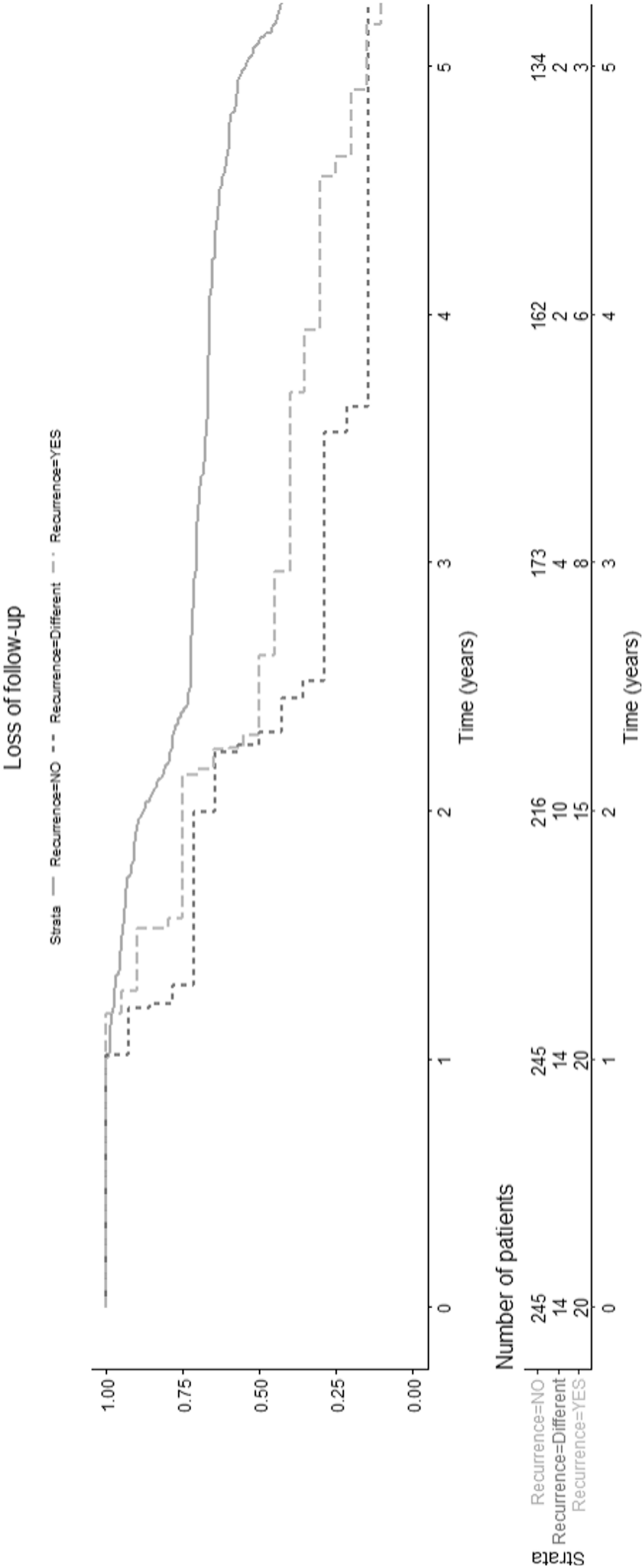
Loss to MRI follow-up. A Kaplan-Meier survival curve showing follow-up duration for study patients up to five years. Stratified by reasons of censoring for loss to follow-up (recurrence, unrelated intracranial tumors or other)

The median time from preoperative MRI to surgery was two weeks (range 0 days to 42.1 weeks). A total of 279 preoperative and 755 postoperative MRI studies were analyzed. The median number of postoperative MRI studies was three per patient (range one to eight). At least two postoperative MRI images were available for 208 (74.6%) patients. The median time from surgery to the first follow-up MRI was 1.3 years (range one day to 10.2 years). Overall, the first postoperative MRI was done within one or two years in 83 (29.7%) and 178 (63.8%) of the patients, respectively. Only 18 (6.5%) patients had the first postoperative MRI after two years. The median recurrence-free follow-up time was 5.0 years (range 1.0 to 19.1 years) (Fig. 3). The follow-up of 20 (7.2%) patients was ended (median follow-up time 2.5 years, range 1.2 to 5.4 years) due to recurrence, and of 14 (5.0%) patients due to other new intracranial tumors (median follow-up time 2.3 years, range 1.0 to 7.8 years) (Fig. 3).

### Intraoperative iatrogenic lesions

Out of 279 patients with preoperative PTBE, only 10 (3.6%) had early in-hospital postoperative MRIs available (Supplementary Table 1). All developed new neurological symptoms and acute ischemic lesions near the surgical site. In nine patients (90%), lesions were outside preoperative PTBE borders, allowing assessment of PTBE resolution; one had lesions within PTBE borders. No distant ischemic lesions were detected. Ischemic tissue volumes ranged from 1.0 to 5.4 cm³ (median 2.9 cm³). Iatrogenic PTBE-like changes were observed in nine (90%) patients around ischemic lesions, with a median volume of 3.52 cm³ (range 0.82 to 16.7 cm³).

### PTBE resolution

**Fig. 4:**
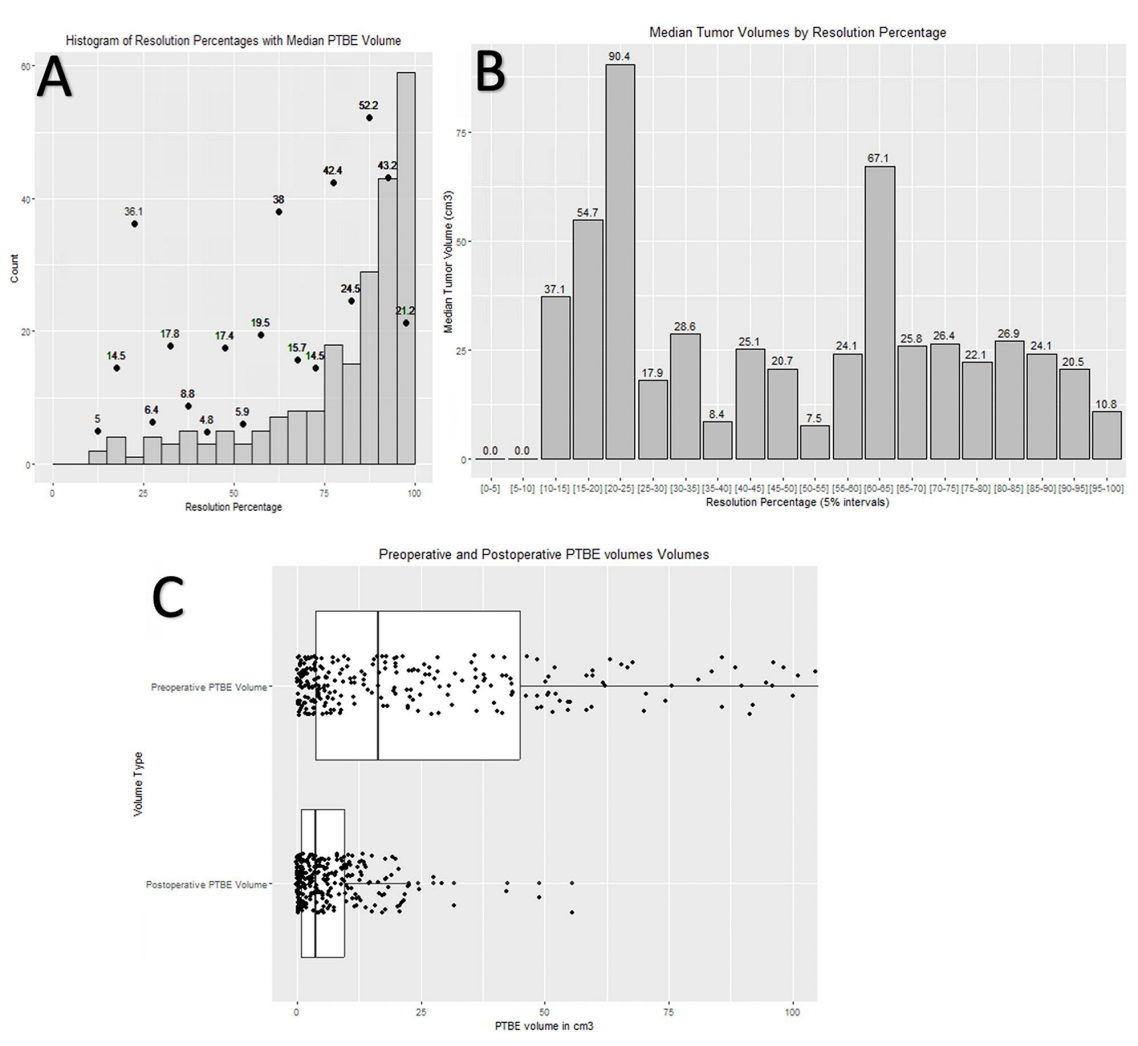
Peritumoral brain edema (PTBE) resolution among 279 patients with preoperative PTBE. (A) Histogram showing resolution percentages among 222 patients with reduction in PTBE. Dots represent median volumes of PTBE for each group. (B) A histogram showing median meningioma volumes for patients in each resolution group. (C) Box plot presenting pre- and postoperative PTBE volumes at the last MRI follow-up

Complete PTBE resolution was observed in only nine (3.2%) out of 279 patients who were MR imaged at one year or later after surgery. In all these cases, PTBE was completely resolved in the first postoperative MRI (median 1.1 years, range 0.6 years to 4.0 years). On the other hand, 25 (30.1%) out of 83, 64 (36.0%) out of 178, and 102 (36.6%) out of 279 patients had ≥ 90% resolution in volume at one year, two years, and last follow-up imaging (median 5.1 years), respectively. The frequency of resolution of ≥ 50% in volume is depicted in Table 2 (Fig. 4A-B). Most (79.6%) patients showed at least some PTBE resolution during the median MRI follow-up time of 5.0 years (Fig. 4C). Ongoing PTBE resolution (≥ 5%) was observed even after 10.4 years, as one patient showed a 5.2% PTBE volume resolution in follow-up MRI taken at 10.4 years; the previous follow-up MRI was taken at 8.5 years. The fastest > 90% resolution occurred in 0.2 years.

**Table 2:** IM patient data by resolution percentage in last MRI follow-up

	Overall	Increased PTBE	0–50% PTBE resolution	> 50% PTBE resolution	> 90% PTBE resolution
Overall, n (%)	279 (100%)	57 (20.4%)	27 (9.7%)	93 (33.3%)	102 (36.6%)
Age in years, median (IR)	59.0 (51.5–67.0)	63.0 (56.0–69.0)	63.5 (57.0–73.0)	59.0 (51.0–66.0)	57.0 (47.0-66.3)
Sex, n (%) Women Men Unspecified	191 (100%) 80 (100%) 8 (100%)	46 (24.1%) 10 (12.5%) 1 (12.5%)	19 (9.9%) 7 (8.8%) 1 (12.5%)	61 (31.9%) 28 (35%) 4 (50%)	65 (34.0%) 35 (43.8%) 2 (25%)
Tumor location, n (%) Skull Base Convexity Parasagittal Falx Other	97 (100%) 94 (100%) 56 (100%) 26 (100%) 6 (100%)	13 (13.4%) 24 (25.5%) 12 (21.4%) 6 (23.1%) 2 (33.3%)	10 (10.3%) 9 (9.6%) 4 (7.1%) 3 (11.5%) 1 (16.7%)	32 (33.0%) 25 (26.6%) 24 (42.9%) 9 (34.6%) 3 (50%)	42 (43.3%) 36 (38.3%) 16 (28.6%) 8 (30.7%)
Tumor laterality, n (%) Right Left Bilateral	128 (100%) 121 (100%) 30 (100%)	24 (18.8%) 28 (23.1%) 5 (16.7%)	10 (7.8%) 13 (10.7%) 4 (13.3%)	40 (31.3%) 38 (31.4%) 15 (50%)	54 (42.2%) 42 (34.7%) 6 (20%)
PTBE location, n (%) Frontal Temporal Parietal Occipital	188 (100%) 49 (100%) 35 (100%) 7 (100%)	33 (17.6%) 10 (20.4%) 12 (34.3%) 2 (28.6%)	20 (10.6%) 2 (4.1%) 4 (11.4%) 1 (14.3%)	73 (38.8%) 10 (20.4%) 7 (20.0%) 3 (42.9%)	62 (33.0%) 27 (55.1%) 12 (34.3%) 1 (14.3%)
Tumor volume in cm^3^, median (IR)	20.2 (8.1–50.4)	25.4 (12.5–56.5)	26.2 (8.9–63.4)	24.6 (8.4–60.4)	13.4 (7.3–29.0)
Tumor area in cm^2^, median (IR)	41.7 (23.3–79.2)	46.7 (29.6–89.9)	47.33 (25.1–89.2)	51.0 (25.4–86.9)	31.3 (20.2–56.8)
Tumor max diameter in cm, median (IR)	4.0 (3.0-5.5)	4.8 (3.5-6.0)	4.3 (3.1–6.1)	4.1 (3.0-5.8)	3.5 (2.7–4.5)
Preoperative PTBE volume in cm^3^, median (IR)	16.4 (3.7–44.9)	1.8 (0.8-3.0)	8.8 (5.1–22.6)	31.3 (9.6–61.6)	25.9 (9.2–57.6)
Preoperative edema index, median (IR)	1.8 (1.2–3.2)	1.0 (1.0-1.1)	1.4 (1.3–1.8)	2.1 (1.5–3.9)	2.9 (1.7–4.9)
Recurrence free follow-up in years, median (IR)	5.0 (2.3–6.5)	4.9 (2.3–5.5)	5.1 (3.0-6.8)	5.0 (2.4–6.4)	5.1 (2.2–7.2)
Postoperative PTBE volume in last MRI in cm^3^, median (IR)	3.8 (0.9–9.6)	6.0 (2.7–10.4)	5.5 (3.6–14.3)	7.0 (2.1–13.0)	0.8 (0.3–2.7)
WHO Grade, n (%) I II	208 (100%) 71 (100%)	42 (20.2%) 15 (21.1%)	19 (9.1%) 8 (11.3%)	74 (35.6%) 19 (26.8%)	73 (35.1%) 29 (40.8%)
Histopathological subtype, n (%) Meningiothelial Atypical Fibrous Transitional Secretory Others Unspecified	86 (100%) 69 (100%) 38 (100%) 38 (100%) 10 (100%) 21 (100%) 17 (100%)	11 (12.8%) 15 (21.7%) 14 (36.8%) 9 (23.7%) 3 (14.3%) 5 (29.4%)	9 (10.5%) 8 (11.6%) 3 (7.9%) 3 (7.9%) 3 (14.3%) 1 (5.9%)	32 (37.2%) 19 (27.5%) 8 (21.1%) 12 (31.6%) 4 (40.0%) 10 (47.6%) 8 (47.1%)	34 (39.5%) 27 (39.1%) 13 (34.2%) 14 (36.8%) 6 (60.0%) 5 (23.8%) 3 (17.6%)
Recurrence, n (%) No Yes Unrelated intracranial tumor	245 (100%) 20 (100%) 14 (100%)	50 (20.4%) 6 (30%) 1 (7.1%)	23 (9.4%) 2 (10%) 2 (14.3%)	84 (34.3%) 3 (15%) 6 (42.9%)	88 (35.9%) 9 (45%) 5 (35.7%)

PTBE = Peritumoral brain edema, IR = Interquartile range, MRI = Magnetic resonance imaging, WHO = World health organization

Higher EI was associated with higher probability of significant > 90% PTBE resolution, with a median PTBE reduction of 76.6% for small (EI < 2), 89.0% for moderate (EI 2–3, *p* <.001) and 93.3% for large (EI > 3, *p* <.001) PTBE (Table 3, Supplementary Table 2). PTBE in the temporal lobe was more likely to resolve significantly (*p* =.018, Supplementary Table 2).

**Table 3:** Summarized patient data for 222 IM patients with (partial) resolution of preoperative PTBE

	Overall	Small PTBE (EI < 2)	Moderate PTBE (EI 2–3)	Large PTBE (EI > 3)
Overall, n (%)	222 (100%)	91 (41.0%)	55 (24.8%)	76 (33.2%)
Age, median (IR)	58.0 (50.0–67.0)	56.5 (46.0–65.0)	56.5 (46.3–67.0)	62.0 (53.5–67.0)
Sex, n (%) Women Men Unidentified	145 (100%) 70 (100%) 7 (100%)	57 (39.3%) 29 (41.4%) 5 (71.4%)	38 (26.2%) 16 (22.9%) 1 (14.3%)	50 (34.5%) 25 (35.7%) 1 (14.3%)
Location, n (%) Skull base Convexity Parasagittal Falx Other	84 (100%) 70 (100%) 44 (100%) 20 (100%) 4 (100%)	30 (35.7%) 32 (45.7%) 21 (47.7%) 6 (30.0%) 2 (50.0%)	21 (25.0%) 15 (21.4%) 13 (29.5%) 4 (20.0%) 2 (50.0%)	33 (39.3%) 23 (32.9%) 10 (22.7%) 10 (50.0%)
Tumor laterality, n (%) Right Left Bilateral	104 (100%) 93 (100%) 25 (100%)	38 (36.5%) 40 (43.0%) 13 (52.0%)	31 (29.8%) 17 (18.3%) 7 (28.0%)	35 (33.7%) 36 (38.7%) 5 (20.0%)
PTBE location, n (%) Frontal Temporal Parietal Occipital	155 (100%) 39 (100%) 23 (100%) 5 (100%)	66 (42.6%) 11 (28.2%) 11 (47.8%) 3 (60.0%)	35 (22.6%) 13 (33.3%) 5 (21.7%) 2 (40.0%)	54 (34.8%) 15 (38.5%) 7 (30.4%)
Tumor volume cm3, median (IR)	19.8 (7.5–48.7)	38.3 (11.2–71.9)	20.16 (11.0-38.9)	9.62 (3.9–21.7)
Preoperative PTBE volume cm3, median (IR)	24.5 (7.9–54.2)	10.3 (4.3–27.3)	31.27 (12.6–57.2)	49.8 (20.9-106.9)
Tumor max diameter cm, median (IR)	4.0 (2.8–5.2)	4.80 (3.6-6.0)	4.0 (3.0–5.0)	3.0 (2.4-4.0)
WHO Grade, n (%) 1 2	166 (100%) 56 (100%)	58 (34.9%) 33 (58.9%)	43 (25.9%) 12 (21.4%)	65 (39.2%) 11 (19.6%)
Histopathology, n (%) Meningiothelial Atypical Transitional Fibrous Secretory Other Unspecified	75 (100%) 54 (100%) 29 (100%) 24 (100%) 10 (100%) 18 (100%) 12 (100%)	25 (33.3%) 32 (59.3%) 14 (48.3%) 8 (33.3%) 1 (10.0%) 9 (50.0%) 2 (16.7%)	23 (30.7%) 11 (20.4%) 4 (13.8%) 9 (37.5%) 5 (27.8%) 3 (25.0%)	27 (36.0%) 11 (20.4%) 11 (37.9%) 7 (29.2%) 9 (90.0%) 4 (22.2%) 7 (58.3%)
Postoperative PTBE volume at last follow-up cm3, median (IR)	2.95 (0.7–9.3)	2.8 (0.6–8.7)	4.0 (0.7–9.2)	2.6 (0.9–10.0)
Resolution percentage %, median (IR)	88.3 (73.4–95.3)	76.6 (52.9–93.1)	89.0 (78.4–94.4)	93.3 (85.9–96.9)
Recurrence free follow-up in years, median (IR)	5.0 (2.4–7.1)	4.6 (2.3–5.5)	5.4 (3.1–9.3)	5.0 (2.4-6.0)

IM = Intracranial meningioma, PTBE = Peritumoral brain edema, EI = Edema index, IR = Interquartile range, WHO = World Health Organization

Sixty-seven (24.0%) of the 279 patients showed increased PTBE in the first postoperative MR images (median 1.1 years, range two days to 3.2 years). Of the 208 patients with at least two postoperative MRI images, 49 (23.6%) showed increased PTBE volumes in the first postoperative MR images (median 1.0 years, range two days to 3.0 years). Of these 49 patients, PTBE resolved partially in 34 (69.4%) and continued increasing in 15 (30.6%) patients, based on subsequent postoperative MRI (median 2.2 years, range 5.4 months to 7.3 years). The PTBE volume increase in 15 patients between the first and second postoperative MRI ranged from 0.1 to 7.5 cm^3^ (median 1.0 cm^3^).

When comparing the resolution of preoperative PTBE to iatrogenic PTBE in nine patients, preoperative PTBE resolved completely in five patients (55.6%) and partially in four patients (44.4%) at the second postoperative MRI (median 1.2 years, range 3.7 months to 2.2 years), whereas iatrogenic PTBE resolved partially in one, increased in one, and remained stable in seven patients. These nine patients were all considered as having persistent PTBE, due to having persisting iatrogenic lesions, despite preoperative PTBE resolving, as all postoperative hyperintensity surrounding the surgical site was defined as persistent PTBE in this study.

## Discussion

Contrary to our hypothesis, preoperative PTBE persisted in nearly all (96.8%) patients at follow-up MRIs taken at least one year after surgery, regardless of histopathology and IM location. In patients imaged postoperatively in hospital, persisting PTBE could be attributed to iatrogenic surgical lesions in a maximum of half of the patients. PTBE increased initially in 67 (24.0%) patients on the first postoperative MRI, and continued increasing in 15 (5.4%) during follow-up, suggesting a pathophysiologically unknown progressive mechanism in some cases.

The frequency of persisting postoperative PTBE in follow-up MRIs has been reported to vary between 10–70% [13, 25–29]. In our study, a considerably higher prevalence was observed. Specifically, > 90% of PTBE resolution was observed in around one-third (36.6%) of the operated IM patients at the last follow-up MRI (median follow-up time of 5.0 years). This figure falls within the range reported in previous studies [13, 25–29], in which the postoperative assessments of persisting PTBE were done at a mean of 0.3 to 3.8 years [13, 25–29] after surgery, and the longest follow-up time was 13.0 years [29]. In our study, the longest follow-up time was 19.1 years. Even after 19 years, some PTBE persisted.

Although vasogenic edema and cerebral gliosis from prolonged tumor compression, as well as other compressive mechanisms, are speculated contributors to PTBE [30], the exact etiology is unclear. Due to similarities in presentation of edema and gliosis on MRI, radiological differentiation is not certain [31]. Some studies suggest that PTBE resolves in most cases [32]. In our study, PTBE resolution was most modest in patients with large IMs and small-volume PTBE (EI 1–2), suggesting that minimal peritumoral changes may result from IM-induced brain compression, leading to gliosis and persistent PTBE-like changes. Our findings support the view that preoperative PTBE is almost always partially persistent and represents more reactive gliosis than vasogenic edema. In line with this view, PTBE volume does not significantly change after glucocorticoid administration in some IM patients [33].

Patients with larger preoperative PTBE volumes and larger IMs have a higher risk of venous infarctions and contusions [34, 35]. In our study, we observed an increased PTBE in nearly one-fourth (24.0%) of patients in the first follow-up MRI, indicating possible iatrogenic changes. There was no significant association between PTBE resolution and tumor size (*p* =.06), but higher preoperative PTBE volume was associated with higher PTBE resolution percentage (*p* =.001). Between the first and second follow-up MRI studies, PTBE continued to increase in one-third (30.6%) of these patients. It has been speculated that reactive gliosis due to surgery or preoperative PTBE [30] may affect neural remodeling. This neural remodeling in turn may lead to tissue damage in perilesion perimeter regions, which may extend for considerable distances from the focal lesions [36]. This expansion represents increasing PTBE-like changes *(i.e. gliosis)* and could explain why PTBE continues to increase in some cases. Anecdotally, we noticed some digitiform protrusions of PTBE in postoperative FLAIR images (not shown), and these protrusions are believed to indicate anterograde axonal degeneration (*i.e. gliosis)* following nerve injury [37].

Our study has a few strengths in comparison to previous studies. The largest previous study of 75 patients focused on skull base IMs [12], whereas we studied the association of nearly all supratentorial IM locations and histopathology groups. No significant differences were observed between skull base, convexity, falcine, and parasagittal IMs. (Supplementary Table 2) We used a strict criterion for persisting PTBE, whereas previous studies have not reported how persisting PTBE was defined. The previous longest mean MRI follow-up was 3.8 years, while our study included patients with a follow-up of a mean of 5.0 years. Our study also assessed the distribution of persistent changes utilizing early in-hospital MRI.

### Limitations

Our study also has a few major limitations worth noting. Early in-hospital MRI studies were performed only in occasions when the patient presented with new neurological symptoms. Therefore, we could not fully assess the role of intraoperative complications in the persistence of PTBE. However, based on the subgroup analysis of a small number of patients with in-hospital MRIs, it is very unlikely that iatrogenic lesions explain the study findings. Despite the large cohort size, the study failed to assess the PTBE resolution of IMs in infrequent locations and belonging to rare histopathological groups. Due to lack of generalized methods for differentiating between IILs and PTBE existing, we may have missed IILs occurring within preoperative PTBE borders. However, we believe it is unlikely, that all remaining hyperintensity within preoperative PTBE borders in immediate in-hospital MRI is caused by IILs.

We were unable to obtain information on pre- or postoperative steroid use for alleviation of PTBE in the study cohort. It has however previously been shown [33], that steroid use does not significantly change PTBE volume in all cases of IM. If data on use of steroids were available, it could possibly show quicker resolution of “true” edema, but not change the amount of persisting PTBE, which likely represents gliosis.

## Conclusion

Persisting peritumoral brain edema—if edema at all—is a very common finding following GTR of IMs. While complete resolution of PTBE seems to be a rare event, considerable resolution of over 90% was observed in more than one-third of patients. Our findings suggest that preoperative PTBE does not represent purely vasogenic and transient edema, but rather gliotic changes.

## Electronic supplementary material

Below is the link to the electronic supplementary material.

Supplementary Material 1

Supplementary Material 2

## Data Availability

No datasets were generated or analysed during the current study.
