# Supplementary material for: Peritumoral edema resolves infrequently in surgically treated patients with intracranial meningioma– a retrospective study of 279 meningioma patients
Source: J Neurooncol. 2025 Mar 6;173(1):83–94. doi: 10.1007/s11060-025-04964-8 (PMC12040978; doi:10.1007/s11060-025-04964-8)
Supplement: Supplementary file 1 — Supplementary Material 1 [file 11060_2025_4964_MOESM1_ESM.docx]

Supplementary Table 1. General characteristics on IM patients who suffered from surgical complications and were imaged with MRI during the hospital stay.

|  | Overall |
| --- | --- |
| Overall, n (%) | 10 (100%) |
| Age in years, median (IR) | 63.00 (53.3-67.0) |
| Sex, n (%)  Women  Men | 7 (70.0%)  3 (30.0%) |
| Tumor location, n (%)  Parasagittal  Falx  Skull base  Convexity  Other | 4 (40.0%)  2 (20.0%)  2 (20.0%)  1 (10.0%)  1 (10.0%) |
| Tumor laterality, n (%)  Right  Left  Bilateral | 5 (50.0%)  4 (40.0%)  1 (10.0%) |
| Tumor volume in cm^3^, median (IR) | 29.3 (18.7-51.4) |
| Tumor area in cm^2^, median (IR) | 52.8 (40.1-93.6) |
| Tumor max diameter in cm, median (IR) | 4.6 (4.1-5.8) |
| Preoperative PTBE volume in cm3, median (IR) | 18.9 (3.2-34.0) |
| Preoperative edema index, median (IR) | 1.4 (1.2-1.8) |
| Postoperative PTBE volume in in-hospital MRI, median (IR) | 24.0 (14.0-34.9) |
| Postoperative PTBE volume in first MRI after first postoperative year in cm^3^, median (IR) | 6.8 (4.5-9.9) |
| WHO Grade, n (%)  I  II | 4 (40.0%)  6 (60.0%) |
| Histopathological subtype, n (%)  Atypical  Fibrous  Meningiothelial | 6 (60.0%)  2 (20.0%)  2 (20.0%) |
| Resolution percentage in %, median (IR) | 49.2 (-38.8-74.8) |

IR = Interquartile range, PTBE = Peritumoral brain edema, MRI = Magnetic resonance imaging, WHO = World health organization
