# Supplementary material for: Peritumoral edema resolves infrequently in surgically treated patients with intracranial meningioma– a retrospective study of 279 meningioma patients
Source: J Neurooncol. 2025 Mar 6;173(1):83–94. doi: 10.1007/s11060-025-04964-8 (PMC12040978; doi:10.1007/s11060-025-04964-8)
Supplement: Supplementary file 2 — Supplementary Material 2 [file 11060_2025_4964_MOESM2_ESM.docx]

Supplementary Table 2. Results of Results of Welch t-test and linear model among all tumors with (partial) resolution of PTBE

| Associated factor | Number of patients analyzed | Median resolution percentage | 95% Confidence interval | p value |
| --- | --- | --- | --- | --- |
|  |  |  |  |  |
| Sex  Women (R)  Men | 145  70 | 79.1  82.8 | -9.4, 2.0 | .21 |
| Location  Skull Base (R)  Convexity  Parasagittal  Falx  Other | 84  70  44  20  4 | 90.4  90.8  86.0  85.4  71.8 | -9.7, 4.0  -10.5, 5.2  -13.1, 7.9  -37.1, 6.1 | .41  .51  .63  .16 |
| Tumor laterality  Right (R)  Left  Bilateral | 104  93  25 | 90.9  87.0  81.7 | -10.0, 2.0  -16.9, 1.8 | .19  .11 |
| PTBE location  Frontal (R)  Temporal  Parietal  Occipital | 155  39  23  5 | 86.2  94.6  91.1  66.0 | 1.5, 16.5  -10.7, 8.0  -27.9, 9.9 | .018  .78  .35 |
| WHO Grade  1 (R)  2 | 166  56 | 80.1  80.2 | -6.9, 6.6 | .96 |
| Histopathology  Meningiothelial (R)  Atypical  Transitional  Fibrous  Secretory  Other | 75  54  29  24  10  18 | 87.3  92.5  87.6  85.6  85.7  81.8 | -7.2, 7.8  -7.6, 10.9  -7.0, 12.8  -1.5, 27.0  -15.2, 6.9 | .94  .73  .56  .08  .46 |
| Edema index  <2  2-3  >3 | 91  55  76 | 76.6  89.0  93.3 | 5.3, 18.5  13.5, 25.5 | <.001  <.001 |
| Recurrence  No (R)  Yes | 195  14 | 80.0  83.9 | -15.9, 8.2 | 0.51 |

PTBE = Peritumoral brain edema, WHO = World Health Organization
